# Prognostic Implications and Immune Landscape Analysis of Necroptosis-associated Gene Signatures in Acute Myeloid Leukemia

**DOI:** 10.7150/jca.113136

**Published:** 2025-07-11

**Authors:** Zongsi Zhu, Bing Li, Ping Li

**Affiliations:** Department of Hematology, Tongji Hospital, Tongji University School of Medicine, Shanghai, China.

**Keywords:** Necroptosis-related genes, Acute myeloid leukemia, Tumor immune microenvironment, Prognostic nomogram, Drug sensitivity analysis

## Abstract

**Background:** Acute myeloid leukemia (AML) remains an incurable hematological malignancy characterized by significant treatment resistance. Necroptosis, a newly recognized form of programmed cell death, has been implicated in tumor development and progression; however, its specific role in AML is not yet fully understood.

**Materials and Methods:** We integrated transcriptomic and clinical data from TCGA and GEO database (GSE37642) to identify differentially expressed necroptosis-related genes (NRGs) between AML and normal samples from GTEx. Consensus clustering was performed to classify AML samples based on NRG expression profiles. Kaplan-Meier survival analysis, GSVA, and ssGSEA were employed to assess survival differences, biological functions, and immune cell infiltration between clusters. Differentially expressed genes (DEGs) identified between NRG clusters underwent LASSO and Cox proportional hazards regression analyses to develop a prognostic risk model. A nomogram integrating age and risk score was constructed and validated in independent cohorts (GSE12417). A nomogram integrating age and risk score was developed. CNV, TMB, immune profiles, and drug sensitivity were also analyzed. Importantly, qRT-PCR was performed using *THP-1* and normal PBMCs to experimentally validate the expression levels of three key NRGs identified by the model (STAT5B, MAP3K7, and BCL2L11).

**Results:** Two distinct NRG clusters were identified. Cluster B exhibited poorer prognosis, higher immune cell infiltration, and enriched signaling pathways, including TGF-β, JAK-STAT, ERBB, MAPK, and VEGF. The developed prognostic nomogram demonstrated robust predictive capability (integrated AUC = 0.645). The high-risk group displayed positive correlations with naive B cells, eosinophils, activated/resting memory CD4^+^ T cells, and CD8^+^ T cells, while negatively associated with memory B cells, resting mast cells, and follicular helper T cells. Drug sensitivity analysis indicated increased sensitivity to Bcl-2 inhibitors, checkpoint kinase inhibitors, and MAPK-MEK pathway inhibitors in the high-risk group. qRT-PCR results confirmed that STAT5B was significantly upregulated, while MAP3K7 and BCL2L11 were significantly downregulated in AML cells compared to normal PBMCs, consistent with bioinformatic predictions.

**Conclusion:** Our study elucidates a significant association between suppressed necroptosis and adverse prognosis in AML. We highlight the role of NRGs in modulating the immune microenvironment of AML and identify potential therapeutic targets and drugs, providing valuable insights for improving clinical outcomes in AML patients.

## Introduction

Programmed cell death (PCD), including apoptosis and necroptosis, is critical for eliminating malignant cells and suppressing oncogenesis 1. Necroptosis is triggered by stimuli such as TNF-α, which binds TNFR1 to activate signaling cascades culminating in cell death 2. TNFR1 engagement recruits TRADD, RIPK1, and RIPK3 to form Complex I (necrosome), where RIPK1 ubiquitination activates pro-survival pathways (NF-κB/MAPK) 3, 4. Within Complex I, RIPK1 activity is a critical regulator; its polyubiquitination mediated by inhibitors of apoptosis (IAP) activates NF-κB and MAPK-ERK signaling pathways 4. If RIPK1 is deubiquitinated (e.g., by CYLD), it dissociates to form Complex IIb with FADD-caspase-8, promoting apoptosis. Conversely, caspase-8 inhibition redirects RIPK1/RIPK3 to phosphorylate MLKL (Complex IIc), executing necroptosis via membrane rupture 5, 6. Thus, FADD-caspase-8 acts as a molecular switch, repressing necroptosis to favor apoptosis 7.

Necroptosis, distinct from apoptosis, triggers immunogenic cell death through DAMPs release and inflammatory cascades 8, yet its dual role in cancer—suppressing tumorigenesis or promoting metastasis—depends on cellular context 9. Key necroptosis regulators (e.g., RIPK3, RIPK1, CYLD) are frequently downregulated across malignancies (colorectal, breast, and hematopoietic cancers) 10-12, enabling tumor immune evasion and aggressiveness. Conversely, FADD overexpression in pancreatic cancer drives tumorigenesis 13, highlighting context-dependent roles of necroptosis machinery. These complex dynamics underscore the dual roles of necroptosis in cancer and the necessity to further elucidate its mechanisms.

Necroptosis further modulates antitumor immunity by regulating dendritic cell (DC) cytokine production and CD8+ T cell activation, critical for immunosurveillance 14. Interestedly, necroptosis can reduce excessive T cells in immune tolerance to reactivate T cell immunity to regulate the antigen-induced proliferation of T cells 15. The initiation of adaptive CD8+ T cell immune responses also heavily depends on RIPK1-mediated NF-κB activation 16. Pharmacological necroptosis induction reshapes the tumor microenvironment (TME) by suppressing Tregs and enhancing cytotoxic CD8+ T cells, demonstrating therapeutic potential 17. Collectively, these findings highlight necroptosis as a crucial regulator of antitumor immunity, underscoring its potential therapeutic relevance in cancer treatment. In acute myeloid leukemia (AML), where chemotherapy resistance and relapse prevail despite multimodal therapies 18, 19, targeting necroptosis—a mechanism to bypass apoptosis resistance—holds untapped therapeutic promise.

In this study, we leveraged publicly available transcriptomic and clinical data from The Cancer Genome Atlas (TCGA) and Gene Expression Omnibus (GEO) databases to comprehensively investigate the association between necroptosis-related genes (NRGs) and AML prognosis. We identified distinct clusters based on NRG expression patterns and assessed their correlations with patient survival and immune microenvironment characteristics. Subsequently, we constructed a prognostic nomogram incorporating key NRGs and clinical features to predict AML patient outcomes. Additionally, we explored differential drug sensitivity profiles between risk groups, aiming to identify potential therapeutic agents specifically targeting high-risk AML subgroups.

## Materials and methods

### Data collection and arrangement

We download the transcriptome and clinical data from TCGA and GEO. All samples with incomplete clinical or survival data were excluded. A total of 142 AML samples from TCGA and 402 samples from the GEO database (GSE37642) were included. Batch effects between the two datasets were corrected using the “removeBatchEffect” function in the “limma” package, after which they were merged into a combined cohort of 544 AML samples. Specifically, we randomly split the 544 samples into training (n = 272) and internal validation cohorts (n = 272) using the “caret” package in R. The training cohort was used to build the prognostic model, while the internal validation cohort assessed its stability. GSE12417 served as an external validation set. In addition, we selected 337 normal blood samples from The Genotype-Tissue Expression (GTEx) as the control group.

Somatic mutation profiles and copy number variation (CNV) data for TCGA AML samples were obtained from the UCSC Xena platform. All subsequent analyses were conducted using R software (version 4.4.1), various R Bioconductor packages, and Perl scripts.

### Identified necroptosis-related genes, TMB and CNV

We retrieved 125 necroptosis-related genes (NRGs) from public databases and previous literature, using the keyword “necroptosis,” and selected genes with a relevance score ≥1.0 to ensure strong biological association 5, 20, 21. Differential expression analysis between AML samples from TCGA and the GTEx control group was performed using the “limma” packages in R. Tumor mutation burden (TMB) analysis was conducted using the “maftools” package in R and custom Perl scripts. Additionally, CNV patterns and chromosome locations of the identified NRGs were visualized using the “Rcircos” package.

### Clustering analysis of NRGs in AML

To acquire consensus NRGs in the entire cohort, we discarded some NRGs that were only present in the TCGA samples. And 109 NRGs were eventually identified as shared genes (Table S1). We used “survminer” packages to perform Cox analysis to find 21 NRGs influencing the survival of AML patients. Then, we constructed protein-protein interaction (PPI) from STRING and a correlation network by the “igraph”, “psych” and “reshape2” packages. Based on 21 NRGs, we used the “ConsensusClusterPlus” package to divide the entire cohort into two NRG clusters. The optimal number of clusters was determined by examining the cumulative distribution function curve and the consensus matrix heatmap. These methods ensured stable and robust clustering results. Kaplan-Meier (K-M) surv ival analysis was subsequently performed between the two clusters using the “survminer” package. We also used the “limma” and “ggplot2” packages to draw a principal component analysis (PCA) plot to test the capacity of NRGs to distinguish the clustering results. To investigate the correlation of biological functions and NRGs, we downloaded “c2.cp.kegg.v7.4.symbols.gmt” from MSigDB. Differentially enriched pathways were visualized through a gene set variation analysis (GSVA) heatmap generated using the “GSVA” package. Furthermore, single-sample gene set enrichment analysis (ssGSEA) using the “GSEAbase” package was performed to evaluate immune cell infiltration levels between clusters.

To explore the potential function of two NRG clusters, we used “clusterProfiler”, “enrichplot” and “org.Hs.eg.db” packages for Gene Ontology (GO) and Kyoto Encyclopedia of Gene and Genomes (KEGG) enrichment analysis and mapped bubble plot.

### Identified the DEGs and established a nomogram

Initially, univariate Cox regression analyses were conducted using the “limma” package to identify differentially expressed genes (DEGs) between the two NRG clusters. Subsequently, LASSO regression and multivariate Cox proportional hazards analyses were performed using the “survminer”, “glmnet” and “timeROC” packages, resulting in the identification of 13 significant DEGs. Based on 13 DEGs, we calculated the risk score of each sample. The risk score calculation formula was as follows: risk score = Coef1 × Exp1 + Coef2 × Exp2 + … + Coef13 × Exp13. Then, we distinguished the entire cohort into high- and low-risk groups based on the median value of the risk score. Meanwhile, we performed the K-M analysis and drew the risk curves and survival status curves of the entire, training and testing cohort by “pheatmap” package. To assess the predictive value, we used the “timeROC” packages to draw the receiver operating curve (ROC) of 1-, 3- and 5 years. In addition, we also performed the same works for the external cohort to validate the accuracy of our study.

Based on DEGs, we used the “ConsensusClusterPlus” package to cluster again and obtained two clusters (DEG cluster A and DEG cluster B). The K-M analysis was also performed by “survminer” package. Finally, to understand the relationship among NRG cluster, DEG cluster, risk score, and survival status, we drew Sankey's diagram by “ggalluvial” and “ggplot2” packages.

Combining with risk score and a clinicopathological feature (Age), we drew the nomogram model, calibration curve, and area under the curve (AUC) to predict the prognosis and survival in the 1-, 3-, and 5- year survival rate by “rms”, “regplot” and “readr” packages.

### Immune infiltration TMB and drug sensitivity between the high- and low-risk groups

To study related immune cells, we used the CIBERSORT algorithm can help us to calculate AML samples in the fraction of 23 immune subsets in high- and low-risk groups 22. We summarize the frequency and somatic mutation of CNV and plot the landscape of genetic alternation and expression variation between high- and low-risk groups. Eventually, we investigated the drug sensitivity between the high- and low-risk groups by “pRRophetic” packages. Using the package, we predicted the half-maximal inhibitory concentration (IC50) values for various chemotherapeutic agents. This strategy underscores the potential of risk-stratified treatment approaches in AML management.

### Quantitative Real-Time PCR Validation

To validate the expression levels of 3 key NRGs (STAT5B, MAP3K7, BCL2L11) identified from our bioinformatics analysis, quantitative real-time PCR (qRT-PCR) was performed. The primer sequences used for STAT5B, MAP3K7, and BCL2L11 were designed based on published literature and verified using NCBI Primer-BLAST. And the primer sequences used for STAT5B, MAP3K7, and BCL2L11 are listed in **Table 1**. The human acute myeloid leukemia cell line THP-1 and peripheral blood mononuclear cells (PBMCs) from healthy donors were used as the tumor and normal control groups, respectively. Both cell types were cultured under standard conditions (RPMI-1640 medium supplemented with 10% fetal bovine serum and 1% penicillin-streptomycin) at 37°C with 5% CO₂.

Total RNA was extracted using the TRIzol reagent (Invitrogen), and reverse transcription was performed with a PrimeScript RT reagent kit (Takara). qRT-PCR was conducted using SYBR Green Master Mix (Thermo Fisher Scientific) on a QuantStudio 5 Real-Time PCR System. The relative expression levels of STAT5B, MAP3K7, and BCL2L11 were normalized to GAPDH using the 2^⁻ΔΔCt^ method. All reactions were performed in triplicate.

## Results

### The mutation landscape and CNV location of NRGs

We obtained 125 NRGs between TCGA AML and normal samples. In addition, we summarized all samples' clinical features of TCGA and GEO databases (**Table 2**). Statistical comparisons revealed significant differences in overall survival time (p <0.001) and survival status (p = 0.014) among the three cohorts. These results indicate potential heterogeneity across datasets that should be considered when interpreting downstream analyses. It is worth noting that sex information was not available in the publicly accessible clinical metadata of the GSE37642 and GSE12417 datasets, and thus could not be included in the comparison.

**Figure 1:**
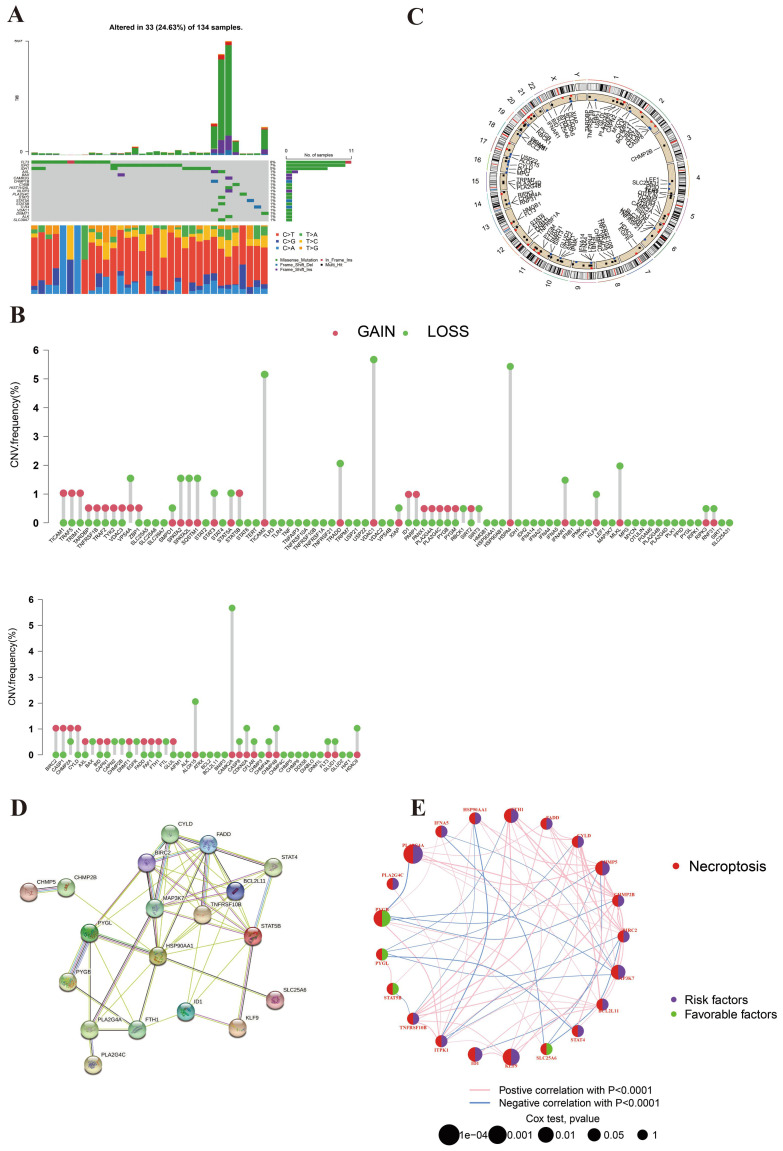
**Genetic mutation landscape of NRGs in AML. (A)** The TMB frequencies of NRGs in 134 AML samples. **(B)** CNV frequencies of NRGs in AML. The mutated frequencies were shown the height. **(C)** Locations of the CNV alteration on chromosomes in NRGs. **(D)** PPI network shown the interaction of NRGs. **(E)** Genes interactions among NRGs in AML. The line connecting of NRGs represents their interaction. Pink and blue represent positive and negative correlations, respectively.

The genetic mutation landscape was shown in **Figure 1A**. This picture suggested that 14 NRGs mutations appeared in 32 of 134 AML samples (23.88%). Among these genes, FLT3 was the most mutated NRG (8%). The mutated frequencies of IDH2 and IDH1 were 7% and 5%, respectively. It indicated the important roles of FLT3 and IDH in the development of AML. As for CNV, the analysis result revealed that the CNV of NRGs in AML was predominantly deletion (**Figure 1B**). CAMK2A, HSPA4, TICAM2, TRADD, and VDAC1were the main deleted genes, which probably indicated that necroptosis was suppressed in AML. Surprisingly, we did not observe significant gained genes in this picture. **Figure 1C** displayed the chromosome location of NRGs. It suggested that NRGs are distributed on nearly every chromosome.

**Figure 1D** illustrates the protein-protein interaction (PPI) network, highlighting potential biological interactions among the 21 identified NRGs, which helps to elucidate the functional relationships underpinning necroptosis pathways in AML. Furthermore, Figure 1E visually depicts the prognostic correlations among these genes, emphasizing their prognostic roles and potential as therapeutic targets. The picture shows that 17 NRGs are favorable factors and 4 PRGs are marked as risk factors.

### Construction and analysis of NRG clusters

**Figure 2:**
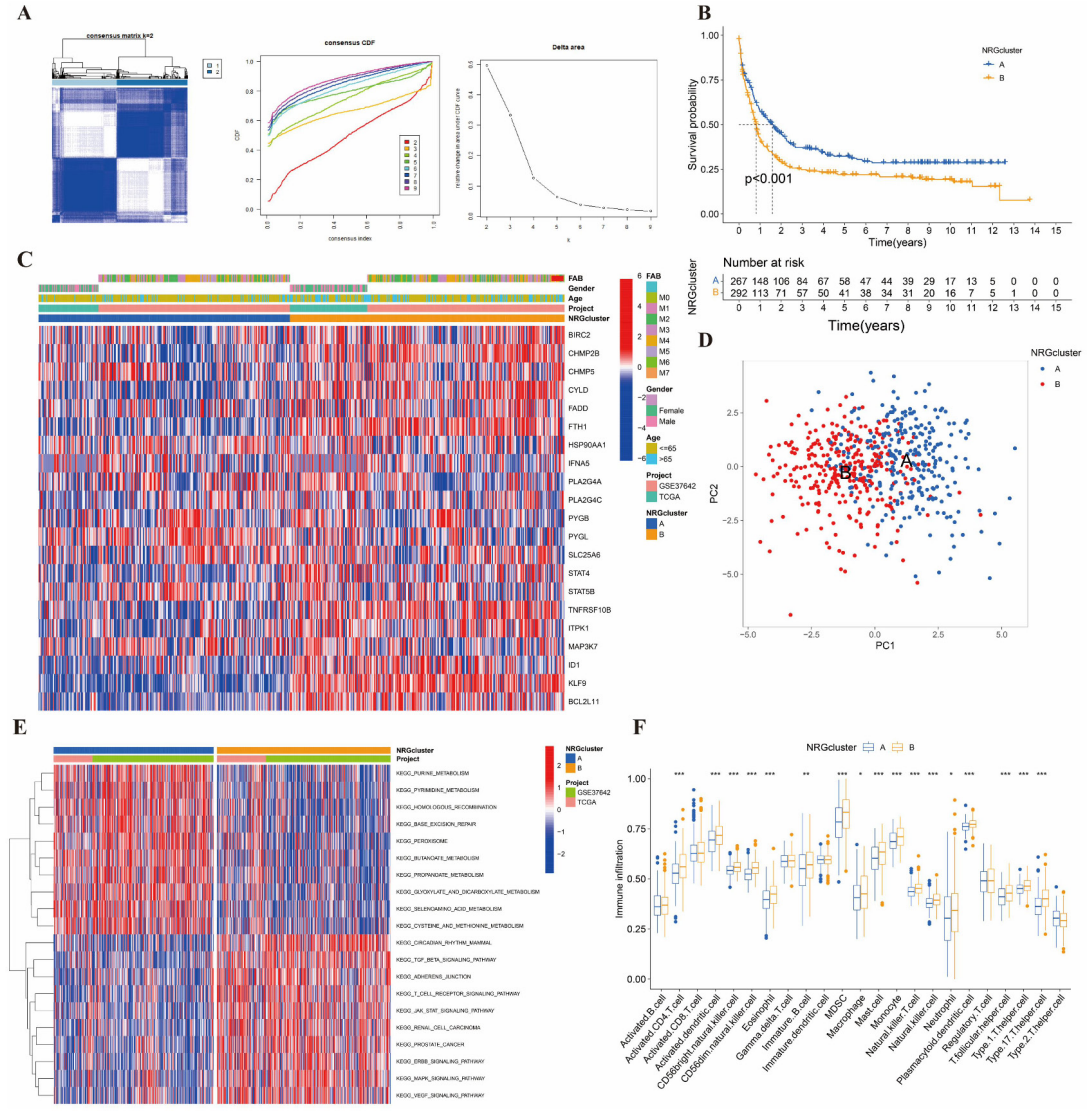
** Identified and analyzed of NRG clusters. (A)** Consensus heatmap of two NRG cluster (k=2). **(B)** The Kaplan-Meier survival analysis of two NRG clusters (p<0.001). **(C)** The heatmap of NRG clusters in clinicopathological features of AML samples. **(D)** PCA analysis in two NRG clusters. **(E)** KEGG pathways analysis in NRG clusters by GSVA. **(F)** Immune cells infiltration between NRG clusters by ssGSEA. *, **, and *** represent p < 0.05, < 0.01, and < 0.001, respectively.

We used the consensus clustering algorithm to divide the entire cohort into two NRG clusters (**Figure 2A**). K-M analysis of two clusters was shown in **Figure 2B**, suggesting they differ in survival time (p<0.001). Cluster B was more associated with poor survival than cluster A. We drew the heatmap to estimate the prognostic values of clusters (**Figure 2C**). PCA analysis indicated two clusters can be easily distinguished (**Figure 2D**).

In addition, we mapped the GSVA and ssGSEA analysis based on 21 NRGs to investigate biological pathways and infiltrated immune cells (**Figure 2E-2F**). In Figure 2E, Cluster A showed enrichment in terms of pathways associated with DNA synthesis and repair, and biochemical material metabolism. In cluster B, the picture showed significant enrichment of growth factors signaling pathways, such as TGFβ, JAK-STAT, ERBB, MAPK, and VEGF signal pathways. As for ssGSEA analysis, we were surprised to find that the 17 differential immune cells all in cluster B, including activated CD4+ T, activated dendritic, CD56bright NK, CD56dim NK, Eosinophils, immature B, MDSC, macrophage, mast, monocyte, NK T, NK, neutrophil, plasmacytoid dendritic, follicular helper T cells (Tfh), and type 1, 17 helper T cells. It suggested that nearly all immune cells upregulated in different degrees in AML patients with poor prognoses.

**Figure 3:**
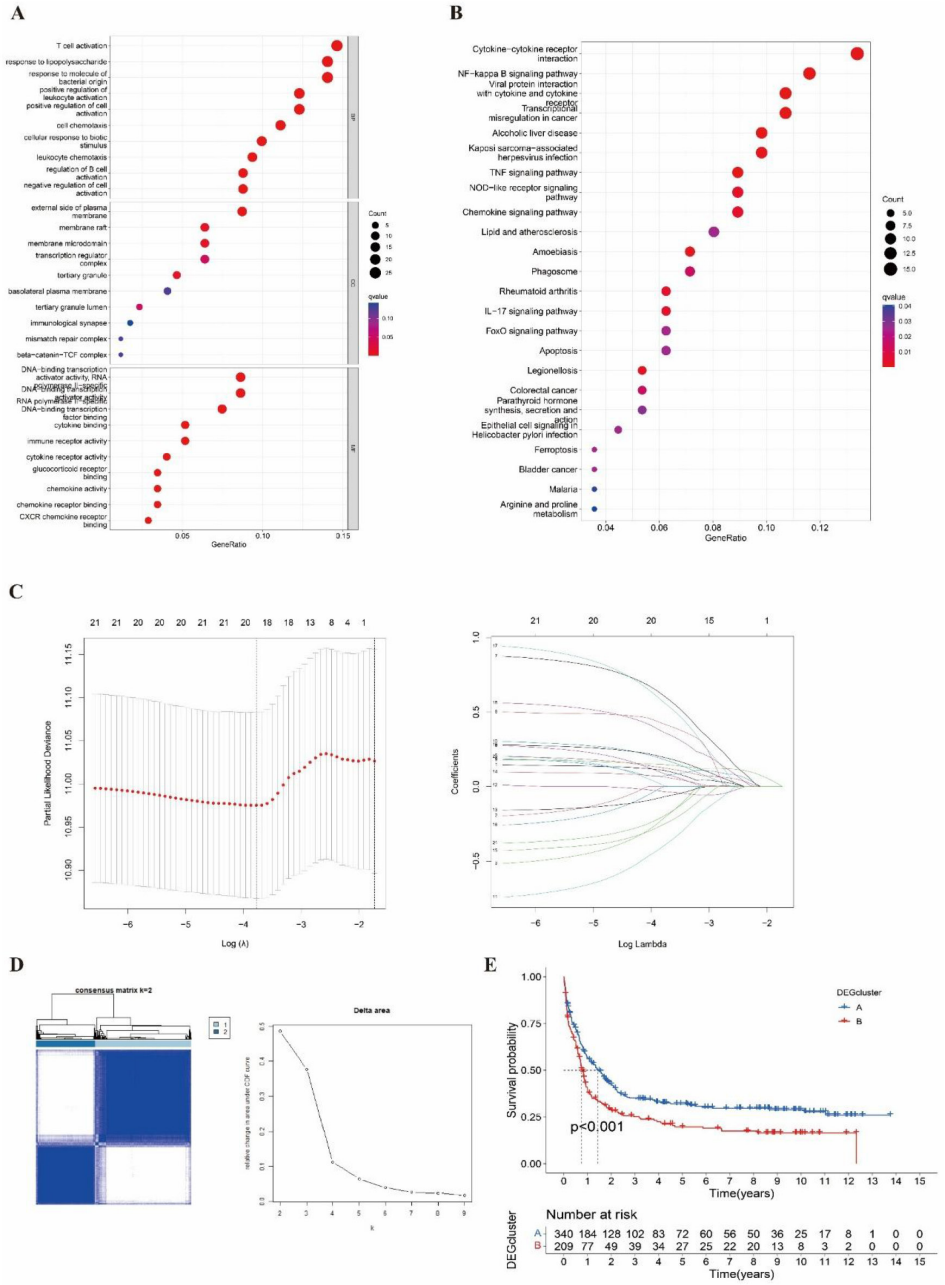
** Identified and analyzed DEGs and DEG clusters. (A-B)** GO and KEGG enrichment analysis between NRG clusters. **(C)** Hazard ration of Cox and LASSO to screen DEGs. **(D)** Consensus heatmap of two DEG cluster (k=2). **(E)** The Kaplan-Meier survival analysis of two DEG clusters (p<0.001).

The GO analysis was shown in **Figure 3A**, which was enriched in T cell activation, lipopolysaccharide and bacterial origin reactions, and leukocyte activation in the biological process (BP). Meanwhile, we performed the KEGG analysis that involved cytokine-cytokine interaction, NK-κB signal pathway, viral protein interaction, and transcriptional dysregulation (**Figure 3B**).

We obtained 13 DEGs (STAT5B, BCL2L11, MAP3K7, BIRC2, CHMP5, HSP90AA1, PLA2G4A, PLA2G4C, PYGB, STAT4, ITPK1, ID1, KLF9) after LASSO and proportional hazard analysis based on NRG clusters (**Figure 3C**). Then, we subdivided the entire cohort into two DEG clusters again and conducted the K-M analysis (**Figure 3D-3E**).

### Identification and Validation of DEGs

**Figure 4:**
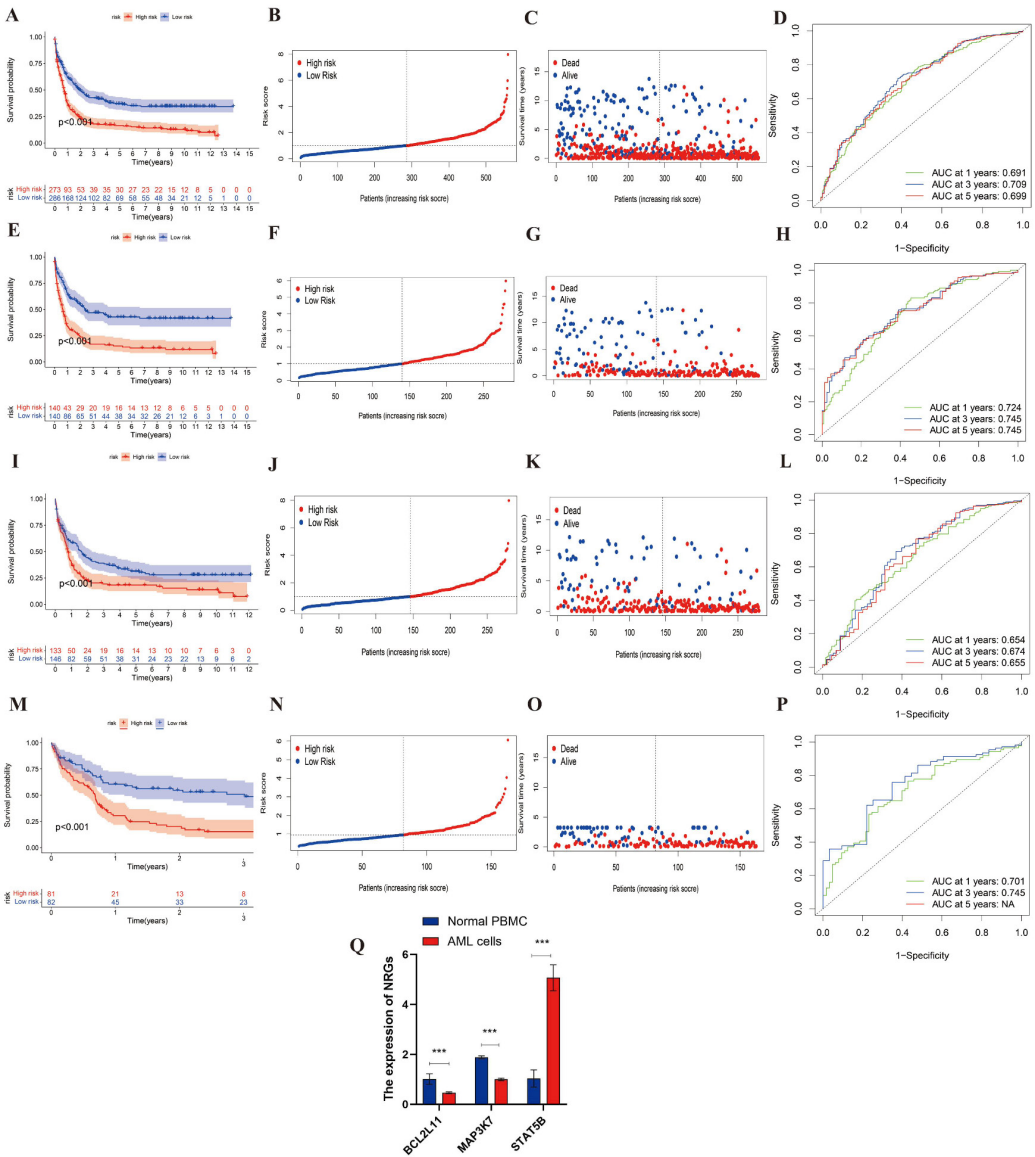
**The evaluation and validation of the prognostic risk score in different AML cohorts and experimental validation by qRT-PCR. (A, E, I, M)** Kaplan-Meier survival analysis of the entire, training, testing, and external validation cohorts, respectively (all p < 0.001). **(B, F, J, N)** Distribution of risk scores in the corresponding cohorts. **(C, G, K, O)** Survival status plots for each cohort, showing the distribution of alive and deceased patients by increasing risk score. **(D, H, L, P)** Time-dependent ROC curves at 1, 3, and 5 years for each cohort, demonstrating the prognostic accuracy of the risk model. **(Q)** Relative mRNA expression levels of BCL2L11, MAP3K7, and STAT5B were validated by qRT-PCR in AML cell line (THP-1) and normal PBMCs. BCL2L11 and MAP3K7 were significantly downregulated, while STAT5B was significantly upregulated in AML cells compared to PBMCs (***p < 0.001). Data are presented as mean ± SEM.

Then, the entire cohort was randomly divided into the training cohort (n = 280) and testing cohort (n = 279). Then, the entire cohort and external validation cohort will be categorized into high- and low-risk after calculating the risk score, which will be used to study the predictive capacity of DEGs. The K-M analysis of the entire, training, testing and validation cohorts were shown in **Figure 4A, 4E, 4I, 4M** (all p<0.001). It indicated an obvious difference between these cohorts in survival. And the risk curve and survival status curve of four cohorts suggested that the high-risk sample can be easily separated from other samples (**Figure 4B-4C, 4F-4G, 4J-4K, 4N-4O**). Moreover, we mapped the ROC to evaluate the prognostic values (**Figure 4D, 4H, 4L,4P**). To be specific, the entire cohort AUCs of 1-, 3- and 5 years were 0.691, 0.709, and 0.699, respectively. As for the training cohort, the AUCs of 1-, 3- and 5 years were 0.724, 0.745, and 0.745, respectively. And the AUCs of the testing cohort was 0.654, 0.674, and 0.655, respectively. In addition, the external cohort with AUCs of 0.701 and 0.745 at 1- and 3 years. Above all, we found that the risk score could be perfectly used to predict the clinical outcome of AML patients.

To confirm the differential expression of the three NRGs identified by LASSO regression, we conducted qRT-PCR in THP-1 and normal PBMC cells. As shown in **Figure 4Q**, STAT5B expression was significantly upregulated in THP-1 cells compared to PBMCs (p < 0.001), consistent with its positive regression coefficient in the risk model. In contrast, MAP3K7 and BCL2L11 were both significantly downregulated in THP-1 cells relative to PBMCs (p < 0.001), corroborating their negative coefficients and potential protective roles. These results further support the predictive value of the identified gene signatures and their association with AML pathogenesis.

### Establishment of a nomogram to predict prognosis

**Figure 5:**
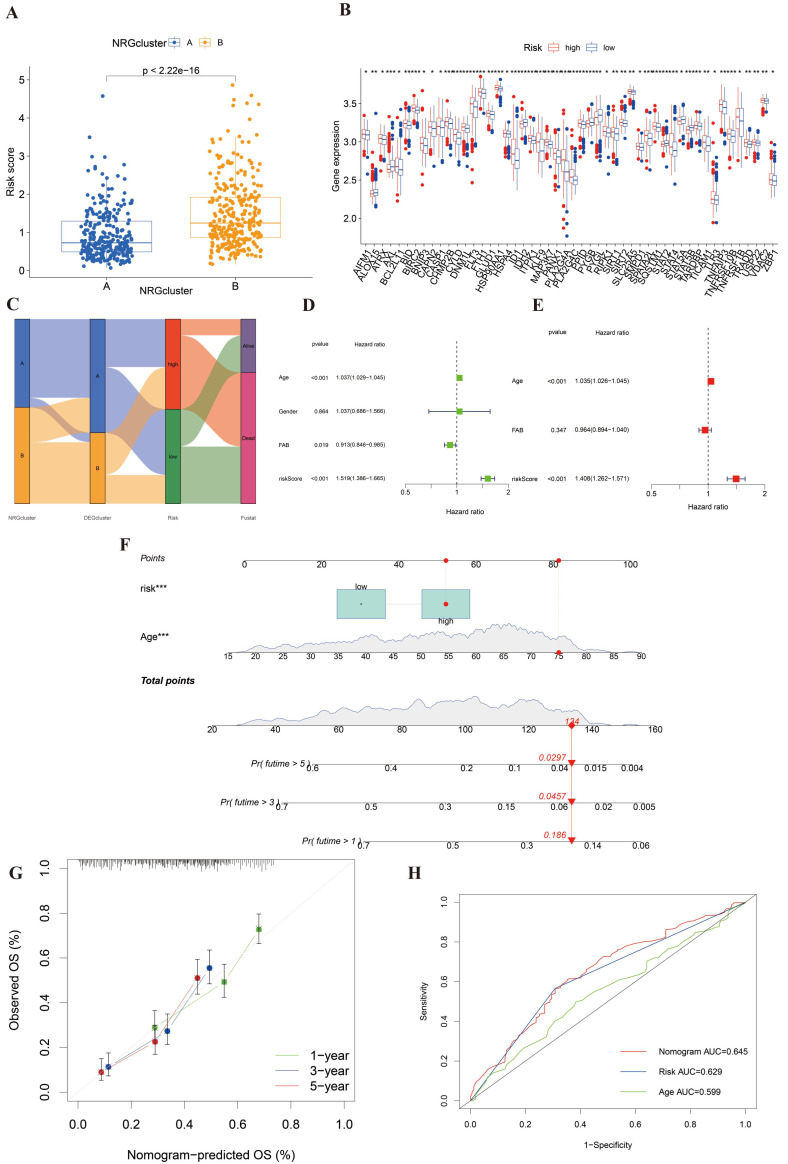
**Established a nomogram to predict the survival of AML patients. (A)** The correlation of risk score and NRG clusters (p< 0.001). **(B)** The significantly differential NRGs between high- and low-risk groups. **(C)** Sankey diagram to display the connection of NRG clusters, DEG clusters, risk score, and clinical outcomes. **(D-E)** Univariant and multivariant Cox analysis of clinicopathological and risk score of AML samples. **(F)** The nomogram integrating age and risk score for AML patients. **(G)** The calibration curve of the nomogram at 1, 3, and 5 years, respectively. **(H)** AUC curve of nomogram, risk score and age. *, **, and *** represent p < 0.05, < 0.01, and < 0.001, respectively.

To construct a connection between NRG clusters and risk groups, we mapped **Figure 5A**. In this picture, we found that the risk score of cluster B was distinctly higher than cluster A. It revealed that cluster B was majorly linked with high risk and poor prognosis. We also found that most of the NRGs were significantly different between high- and low-risk groups after differential analysis (**Figure 5B**). Besides, the relationship among NRG clusters, DEG clusters, risk score, and clinical outcomes was shown in **Figure 5C**. We observed that the patients of NRG cluster B majorly linked to DEG cluster B and high-risk score, which indicated that they were correlated with poorest clinical outcome.

We selected three clinical data (age, gender, and FAB subtypes) and the risk score of the entire cohort as the prepared factors. Only age and risk score were included in the final model because they were the only variables that remained statistically significant in both univariate and multivariate Cox regression analyses. Thus, we finally identified age and risk score as independent prognostic factors to establish a nomogram after univariate and multivariant Cox regression analysis (**Figure 5E-5F**).

We constructed the nomogram including age and risk score to predict the clinical survival of AML patients. Thus, we drew calibration curves to assess the accuracy of nomogram for predicting 1-, 3- and 5-years survival outcomes (Figure 5G). The comprehensive AUC was 0.645 and the predictive capacity of the nomogram is better than that of any single factor (Figure 5H). It indicated that this model might be suitable for AML.

### Immune cell infiltration, mutation, drug sensitivity between high- and low-risk groups

**Figure 6:**
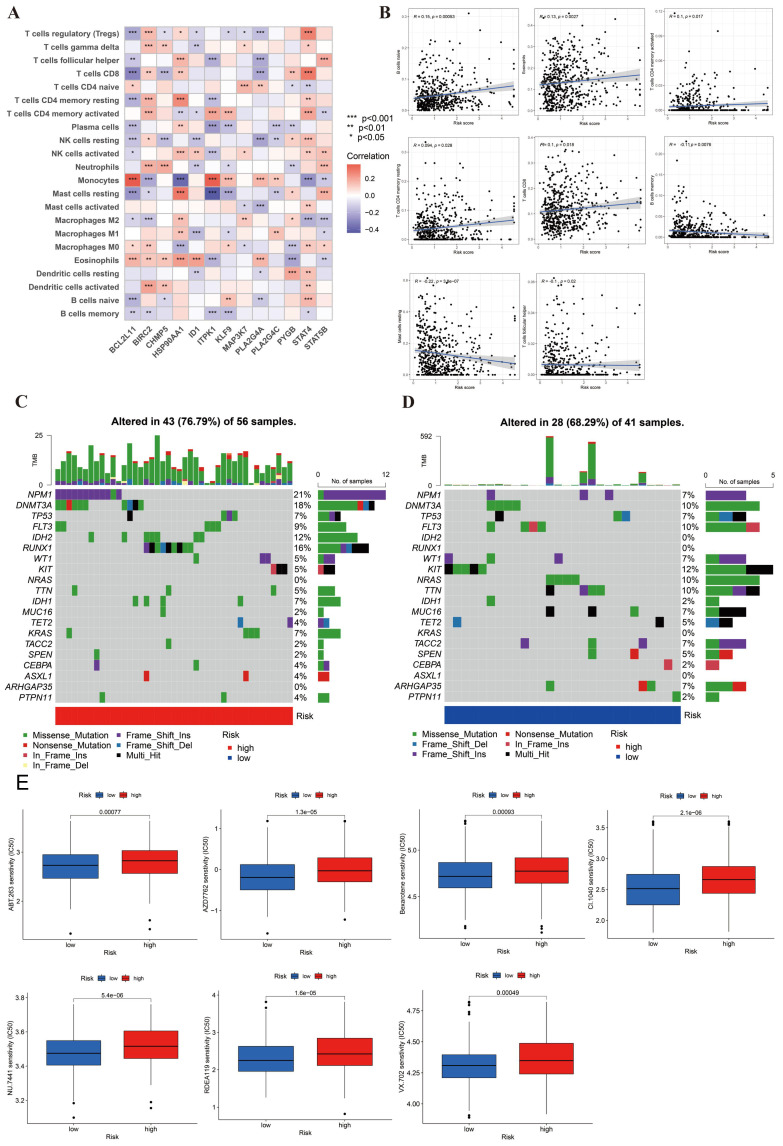
**Identification of immune cells infiltration, genetic variants, and drug sensitivity between high- and low-risk groups. (A)** The infiltration abundance of immune cells in DEGs. **(B)** 8 differential immune cells were correlated with risk score. Positively correlated cells included naive B cell, eosinophils, activated and resting CD4+ T memory cells, and CD8+ T cells. Negatively correlated cells included B memory cells, resting B cells and follicular helper T cells (p < 0.05). **(C-D)** The CNV frequencies in high- and low-risk group. **(E)** 7 sensitive drugs in high-risk group. These drugs were ABT.263, AZD7762, Bexarotene, CI.1040, NU.7441, RDEA119, VX.702, respectively (p < 0.001). *, **, and *** represent p < 0.05, < 0.01, and < 0.001, respectively.

In **Figure 2F**, we explored the relationship between 17 immune cells and NRG clusters. However, our research needed to further define the relationship between DEGs and immune cells. We investigated the infiltration of 22 types of immune cells in DEGs and presented the results in **Figure 6A**. The picture suggested that 5 immune cells have the highest infiltration ratio in 13 DEGs, respectively Tregs (9/13, 69.23%), resting NK cells (8/13, 61.54%), resting mast cells (8/13, 61.54%), macrophage 0 (M0, 8/13, 61.54%), and Eosinophils (9/13, 69.23%). In addition, we screened out 8 differential immune cells in high- and low-risk groups (**Figure 6B**). Immune cells positively correlated with risk score were naive B cells, Eosinophils, activated/resting CD4+ T memory cells, and CD8+ T cells, while B memory cells, resting mast cells and Tfh were negatively correlated.

**Figure 6C-6D** displayed the mutated frequencies of CNV in high- and low-risk groups. Figure 6C indicated that there were 43 mutations in 56 samples (76.79%) in the high-risk group, among which NPM1 (21%), DNMT3A (18%), RUNX1 (16%), and IDH2 (12%) gene mutation frequencies were the highest. As for the low-risk group, KIT had the highest mutation (12%), followed by DNMT3A (10%), FLT3 (10%), NRAS (10%), and TTN (10) with 28/41 (68.29%) altered samples. We saw that frameshift and missense mutations were the main types of mutations in both risk groups.

Lastly, in order to analyze the drug sensitivity in AML cells, we used IC50 drug data from “pRRophetic” package. We obtained the 13 differential results of drug sensitivity in two risk groups (Figure 6E, S1). In this figure, we found that 7 of the 13 drugs were more sensitive in the high-risk group, including ABT.263, AZD7762, Bexarotene, CI.1040, NU.7441, RDEA119, VX.702. These drugs offered potential therapeutic targets for AML.

## Discussion

Chemotherapy remains the cornerstone of AML treatment, primarily acting through apoptosis induction; however, despite long-standing reliance on cytarabine and anthracycline-based regimens, overall prognosis remains unsatisfactory due to frequent relapse and resistance 23. These failures are often attributed to apoptosis dysfunction at the cellular level, underscoring the urgent need to identify alternative non-apoptotic mechanisms of cell death 24. Necroptosis, a regulated form of necrotic cell death, has emerged as a promising compensatory pathway, shown to exert diverse biological effects in solid tumors, though its role in AML has been far less explored 25. Our findings align with previous studies that have highlighted the dual roles of necroptosis in cancer biology. Culver-Cochran AE et al. demonstrated that necroptosis activation enhances the efficacy of chemotherapy in acute leukemia by overcoming apoptosis resistance 26. Similarly, Zhu et al. reported that RIPK3-mediated necroptosis improved immune infiltration in AML cell 27. In contrast, our study expands on these findings by establishing a comprehensive nomogram integrating necroptosis-related gene signatures with clinical parameters, further enhancing AML risk stratification and guiding therapeutic decisions.

We observed a significantly elevated expression of FADD in AML samples compared to the normal controls, supporting its pivotal inhibitory role in the necroptotic cascade 28. Consistent with this, studies in murine models demonstrated that the FADD-caspase-8 complex can block RIPK1-mediated signaling, thereby suppressing necroptosis 29. In addition to its role in necroptosis, FADD has been implicated in promoting tumorigenesis by regulating cell cycle progression, enhancing proliferation, and inhibiting apoptosis, making it a tumor-favoring factor. For instance, in hepatocellular carcinoma, FADD-driven inflammation suppresses the NF-κB pathway, triggering compensatory proliferation and contributing to malignant progression 30. Similarly, in oral squamous cell carcinoma, elevated FADD expression and gene amplification were both associated with advanced tumor stage and unfavorable prognosis 31, 32. Notably, FADD-mediated death receptor pathways have been identified as potential targets for CAR-T therapies in B-cell acute lymphoblastic leukemia 33. Therefore, we assumed that high expression of FADD was closely related to poor prognosis in AML since FADD inhibited necroptosis and developed AML cell proliferation. Meanwhile, molecular drugs or CAR-T targeting FADD was probably a promising therapy to eliminate AML.

We applied unsupervised clustering based on 21 prognostic NRGs and identified two distinct molecular subtypes of AML with divergent clinical outcomes. Cluster B exhibited markedly poorer prognosis and elevated infiltration of immune cells with active phenotypes, including antigen-presenting cells, T/B cells, and cytotoxic NK cells. Interestingly, this cluster also showed significant enrichment of oncogenic signaling pathways such as MAPK and JAK-STAT, both of which are implicated in leukemogenesis and immune resistance. Specifically, MAPK overactivation promotes proliferation and survival in relapsed AML, while JAK-STAT signaling facilitates cytokine-mediated expansion and immune evasion in hematologic malignancies 34. These molecular features likely contribute to the inferior survival of Cluster B patients and underscore potential targets for therapeutic intervention in high-risk subgroups. In addition, 13 DEGs were identified between clusters, and their derived risk score demonstrated strong predictive power for clinical outcomes, further supporting their prognostic relevance.

Clinically, the constructed nomogram provides a valuable tool for stratifying AML patients and informing individualized therapeutic strategies. High-risk patients identified by the model may benefit from intensified treatment, close monitoring, or inclusion in clinical trials of targeted agents, while low-risk individuals could avoid overtreatment and associated toxicities. Furthermore, our drug sensitivity analysis suggests that BCL-2 and MAPK-MEK inhibitors may serve as effective therapeutic options for high-risk groups, reinforcing the utility of risk-based treatment approaches.

Our study revealed the complexity of the AML's TME, with elevated infiltration of immune cells (Tregs, M2 macrophages) in Cluster B correlating with leukemic cell survival. Despite chemotherapy as the frontline treatment, over two-thirds of AML patients exhibit refractory disease 35. This underscores the need for novel strategies, including immunotherapy targeting leukemic cells and progenitors. However, TME-driven immune evasion contributes to treatment resistance 36, with high-risk AML characterized by infiltrating immunosuppressive populations (Tregs, M0 macrophages) linked to DEG activity. As for infiltrated abundance, the relationship between DEGs and immune cells suggested that the B cell, Tregs cells, resting NK cells, resting mast cells, macrophage 0, and Eosinophils were dense and linked to the high-risk prognosis. While B cells may enhance checkpoint blockade efficacy in solid tumors 37, their role in AML diverges, as DEGs promote immunosuppressive niches. In addition, the B cell-related genes were expressed in patients who were sensitive to ICB compared to non-counterparts 38. Tregs, known to suppress antitumor immunity across malignancies 39, 40, are enriched in AML and associated with disease progression. Thus, targeting Tregs may disrupt the immunosuppressive TME dominated by these cells and MDSCs, offering a therapeutic avenue for high-risk AML. Unlike solid tumors, AML TME paradoxically associates heightened immune activity with adverse outcomes. This immune contexture likely enables leukemic escape by impairing cytotoxic responses, explaining the prognostic paradox observed.

Lastly, we performed the drug sensitivity analysis and found that the inhibitor of BCL-2, inhibitor of checkpoint kinase (Chk), and the inhibitor of MAPK-MEK pathways were mainly objective drugs. ABT.263 (Navitoclax) is a targeted inhibitor of BCL-2, which can induce cancer cell to apoptosis. Its similar drug, venetoclax, had been approved to treat AML. The major function of Chk was initiating the DNA damage response to repair impaired DNA. Activated Chk was an important feature in cancer, so its inhibitor could be the ideal target to treat tumors. AZD7762 is an inhibitor of Chk and it will be a potential therapeutic drug for AML. In addition, we found that the MAPK-MEK signaling pathway was enriched in cluster B in GSVA analysis. It indicated that the inhibitor of this pathway is also the crucial therapeutic orient for AML. Meanwhile, these drugs, CI.1040, NU.7441, RDEA119, and VX.702, were exactly consistent with the above discussion. But the correlation between necroptosis and these drugs is unclear, needing subsequent studies.

It should be noted that this work certainly has many flaws. Firstly, all analyses were based on data downloaded from public databases, and all samples used in our study were obtained retrospectively. Thus, selective bias is inevitable. Moreover, we should use more independent AML cohorts in the future to improve the accuracy of prognostic models. And large prospective studies and additional *in vivo* and *in vitro* studies are needed to confirm our findings. In addition, other clinical data, like gender, and stages, is unavailable, which may affect the prognostic estimating.

## Conclusion

In summary, our study establishes a prognostic nomogram based on necroptosis-related gene signatures and clinical parameters, demonstrating significant predictive accuracy for acute myeloid leukemia patient outcomes. Our findings indicate that downregulation of NRGs is strongly associated with poorer prognosis in AML. This research provides valuable insights into the role of necroptosis in AML pathogenesis, highlights potential therapeutic targets, and offers a reliable prognostic tool for clinicians, ultimately contributing to improved management strategies and clinical outcomes for AML patients.

## Supplementary Material

Supplementary figure and table.

## Figures and Tables

**Table 1:** qPCR Primer Sequence.

Primer name	Sequence (5'-3')
STAT5B-Forward	GCCACTGTTCTCTGGGACAATG
STAT5B-Reverse	ACACGAGGTTCTCCTTGGTCAG
MAP3K7-Forward	CAGAGCAACTCTGCCACCAGTA
MAP3K7-Reverse	CATTTGTGGCAGGAACTTGCTCC
BCL2L11-Forward	CAAGAGTTGCGGCGTATTGGAG
BCL2L11- Reverse	ACACCAGGCGGACAATGTAACG

**Table 2:** The clinical characteristics of studied datasets.

	GSE37642	TCGA AML	GSE12417	P value
Sample counts	402	142	162	
Age, years mean (SD)	54.57 (14.90)	54.39 (16.34)	55.63 (14.88)	
Gender				
Male	-	78	-	
Female	-	64	-	
FAB subtypes				
M0	14	-	5	
M1	84	-	45	
M2	117	-	45	
M3	19	-	0	
M4	104	-	42	
M5	47	-	19	
M6	15	-	6	
M7	2	-	0	
OS				< 0.001***
Time, years mean (SD)	2.84 (3.74)	1.57 (1.64)	1.25 (1.16)	
Survival status				0.014*
Dead	295	89	103	
Alive	107	53	59	

OS, overall survival; SD, standard deviation. p-values for OS time were calculated using one-way ANOVA, and for survival status using 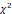
test. *, **, and *** represent p < 0.05, < 0.01, and < 0.001, respectively.
