# Supplementary material for: Prognostic Implications and Immune Landscape Analysis of Necroptosis-associated Gene Signatures in Acute Myeloid Leukemia
Source: J Cancer. 2025 Jul 11;16(10):3202–15. doi: 10.7150/jca.113136 (PMC12305588; doi:10.7150/jca.113136)

Supplementary Table 1 The list of 109 shared NRGs.

ID	Type	ID	Type
AIFM1	necroptosis	STAT5B	necroptosis
ALOX15	necroptosis	STAT6	necroptosis
BAX	necroptosis	TICAM1	necroptosis
BCL2	necroptosis	TLR3	necroptosis
BID	necroptosis	TLR4	necroptosis
BIRC2	necroptosis	TNF	necroptosis
CAMK2A	necroptosis	TNFAIP3	necroptosis
CAPN1	necroptosis	TNFRSF10B	necroptosis
CAPN2	necroptosis	TNFRSF1A	necroptosis
CASP1	necroptosis	TRADD	necroptosis
CASP8	necroptosis	TRAF2	necroptosis
CFLAR	necroptosis	TRAF5	necroptosis
CHMP2A	necroptosis	TYK2	necroptosis
CHMP2B	necroptosis	USP21	necroptosis
CHMP5	necroptosis	VDAC1	necroptosis
CHMP6	necroptosis	VDAC2	necroptosis
CYLD	necroptosis	VDAC3	necroptosis
DNM1L	necroptosis	VPS4A	necroptosis
FADD	necroptosis	VPS4B	necroptosis
FAF1	necroptosis	XIAP	necroptosis
FTH1	necroptosis	ZBP1	necroptosis
FTL	necroptosis	USP22	necroptosis
GLUD1	necroptosis	ITPK1	necroptosis
GLUD2	necroptosis	SIRT3	necroptosis
GLUL	necroptosis	TNFRSF1B	necroptosis
HMGB1	necroptosis	PANX1	necroptosis
HSP90AA1	necroptosis	MAP3K7	necroptosis
HSP90AB1	necroptosis	DIABLO	necroptosis
IFNA14	necroptosis	DNMT1	necroptosis
IFNA21	necroptosis	AXL	necroptosis
IFNA4	necroptosis	ID1	necroptosis
IFNA5	necroptosis	CDKN2A	necroptosis
IFNAR1	necroptosis	HSPA4	necroptosis
IFNB1	necroptosis	FLT3	necroptosis
PARP1	necroptosis	HAT1	necroptosis
PLA2G4A	necroptosis	SIRT2	necroptosis
PLA2G4C	necroptosis	SIRT1	necroptosis
PPID	necroptosis	PLK1	necroptosis
PYGB	necroptosis	MPG	necroptosis
PYGL	necroptosis	MYCN	necroptosis
PYGM	necroptosis	ALK	necroptosis
RBCK1	necroptosis	ATRX	necroptosis
RIPK1	necroptosis	TERT	necroptosis
SLC25A31	necroptosis	SLC39A7	necroptosis
SLC25A5	necroptosis	IDH1	necroptosis
SLC25A6	necroptosis	IDH2	necroptosis
SMPD1	necroptosis	KLF9	necroptosis

SPATA2	necroptosis
SPATA2L	necroptosis
SQSTM1	necroptosis
STAT2	necroptosis
STAT3	necroptosis
STAT4	necroptosis
STAT5A	necroptosis
HDAC9	necroptosis
LEF1	necroptosis
BNIP3	necroptosis
BCL2L11	necroptosis
EGFR	necroptosis
DDX58	necroptosis
TARDBP	necroptosis
TNFRSF21	necroptosis

Supplementary Figure 1 The sensitive drugs between high- and low-risk groups.

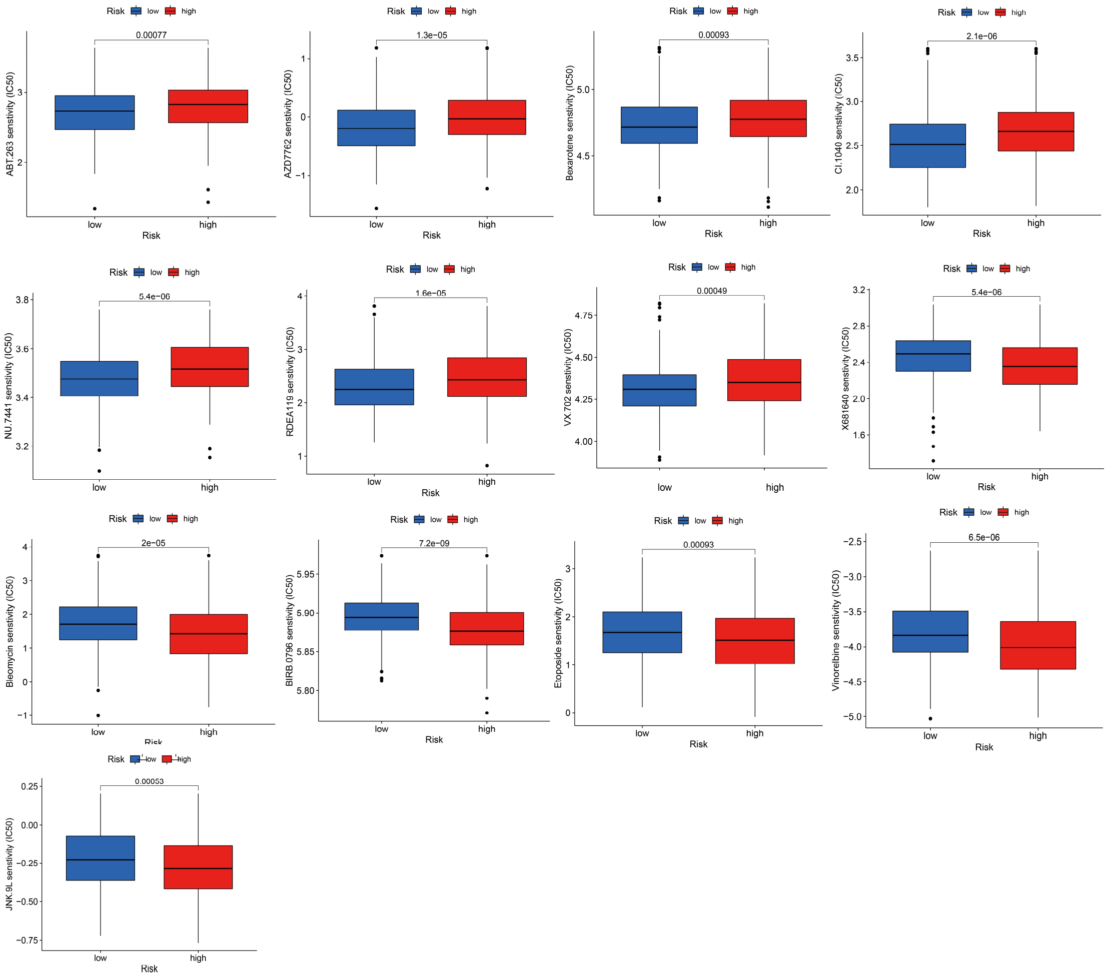

Supplement: Supplementary file 1 — Supplementary figure and table. [file jcav16p3202s1.pdf]
